# A novel 3D deep learning model to automatically demonstrate renal artery segmentation and its validation in nephron-sparing surgery

**DOI:** 10.3389/fonc.2022.997911

**Published:** 2022-10-14

**Authors:** Shaobo Zhang, Guanyu Yang, Jian Qian, Xiaomei Zhu, Jie Li, Pu Li, Yuting He, Yi Xu, Pengfei Shao, Zengjun Wang

**Affiliations:** ^1^ Department of Urology, The First Affiliated Hospital of Nanjing Medical University, Nanjing, China; ^2^ Key Laboratory of Computer Network and Information Integration, Southeast University, Ministry of Education, Nanjing, China; ^3^ Department of Radiology, The First Affiliated Hospital of Nanjing Medical University, Nanjing, China

**Keywords:** tridimensional kidney perfusion model, automatic segmentation, deep learning technique, convolutional neural network, nephron-sparing surgery

## Abstract

**Purpose:**

Nephron-sparing surgery (NSS) is a mainstream treatment for localized renal tumors. Segmental renal artery clamping (SRAC) is commonly used in NSS. Automatic and precise segmentations of renal artery trees are required to improve the workflow of SRAC in NSS. In this study, we developed a tridimensional kidney perfusion (TKP) model based on deep learning technique to automatically demonstrate renal artery segmentation, and verified the precision and feasibility during laparoscopic partial nephrectomy (PN).

**Methods:**

The TKP model was established based on convolutional neural network (CNN), and the precision was validated in porcine models. From April 2018 to January 2020, TKP model was applied in laparoscopic PN in 131 patients with T1a tumors. Demographics, perioperative variables, and data from the TKP models were assessed. Indocyanine green (ICG) with near-infrared fluorescence (NIRF) imaging was applied after clamping and dice coefficient was used to evaluate the precision of the model.

**Results:**

The precision of the TKP model was validated in porcine models with the mean dice coefficient of 0.82. Laparoscopic PN was successfully performed in all cases with segmental renal artery clamping (SRAC) under TKP model’s guidance. The mean operation time was 100.8 min; the median estimated blood loss was 110 ml. The ischemic regions recorded in NIRF imaging were highly consistent with the perfusion regions in the TKP models (mean dice coefficient = 0.81). Multivariate analysis revealed that the feeding lobar artery number was strongly correlated with tumor size and contact surface area; the supplying segmental arteries number correlated with tumor size.

**Conclusions:**

Using the CNN technique, the TKP model is developed to automatically present the renal artery trees and precisely delineate the perfusion regions of different segmental arteries. The guidance of the TKP model is feasible and effective in nephron-sparing surgery.

## Introduction

As a minimally invasive nephron-sparing surgery, laparoscopic partial nephrectomy (laparoscopic PN) is a mainstream treatment for cT1a renal tumors (1). In laparoscopic PN, renal artery clamping induces warm ischemic injury (WII) (2), which could be minimized by segmental renal artery clamping (SRAC) technique, converting global parenchymal ischemia to regional ischemia (3–6). To implement the SRAC technique, dual-source computed tomography (DSCT) angiography was applied to reveal a high-quality three-dimensional vasculature model of the renal hilum, and identify the target segmental arteries needed to be clamped if their branches enter or abut the tumor (6, 7). However, DSCT angiography is unable to provide the details of the perfusion regions of different segmental arteries, and the determination of the target arteries is inaccurate occasionally, which could lead to insufficient clamping and result in arterial bleeding (5). Therefore, a more precise clamping strategy is required.

Based on the contrast CT scan, organ segmentation with different kinds of statistical models were reported in several abdominal organs over the years (8–10). Previously, in our center, to meet the requirement of a more precise SRAC technique, a novel functional three-dimensional perfusion model was established to determine the target arteries by depicting the parenchymal perfusion regions of different segmental arteries using the semi-automatic segmentation of the kidney and renal arteries (11). Recently, with the development of medical image processing technology, convolutional neural network (CNN) as a kind of deep learning technique has gradually begun to be applied in the segmentation of organs and vasculature (12). Based on the CNN, we previously created a series of novel methods, which could provide a fully automatic segmentation of kidney, tumor, and renal artery trees (13, 14). In present study, integrating CNN technique and the distance transformation algorithm, a novel three-dimensional perfusion model was established, which was called the tridimensional kidney perfusion (TKP) model.

## Materials and methods

The establishment procedure of the TKP model was presented. The accuracy of the TKP model was verified in porcine models, and the feasibility and efficacy of this model were assessed in patients undergoing laparoscopic PN with SRAC.

### Establishment of the TKP model

#### Automatic segmentation of the kidneys and tumors

**Figure 1:**
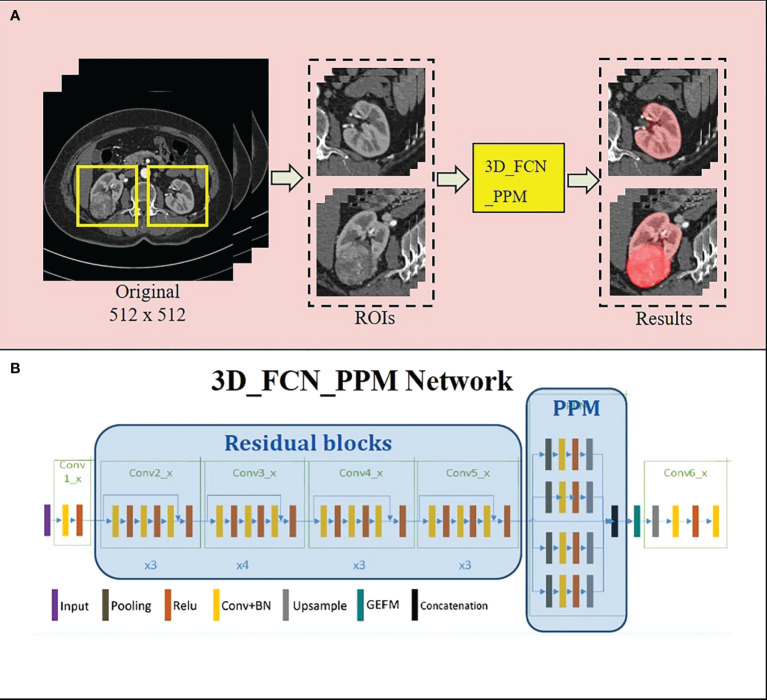
The 3D_FCN_PPM network is applied in the automatic segmentation of kidney and tumor. **(A)** the pipeline of kidney and tumor segmentation; **(B)** the architecture of the 3D_FCN_PPM network.

Our homemade three-dimensional fully-supervised convolutional neural (FCN) network with a pyramid-pooling module (PPM) (3D_FCN_PPM) was proposed for segmentation of kidneys and tumors previously (13) (**Figure 1**). During the establishment of the 3D_FCN_PPM network, the abdominal CT images of 140 patients were recruited from the department of radiology after the informed consent was obtained. And the images were obtained and analyzed in Dicom format. Ninety images were used for the training set, and the remaining 50 images were used for testing. The 3D_FCN_PPM network was demonstrated to be efficient and precise in segmentation with the dice coefficient equal to 0.931 for kidney and 0.802 for renal tumors.

#### Automatic segmentation of the renal artery trees

**Figure 2:**
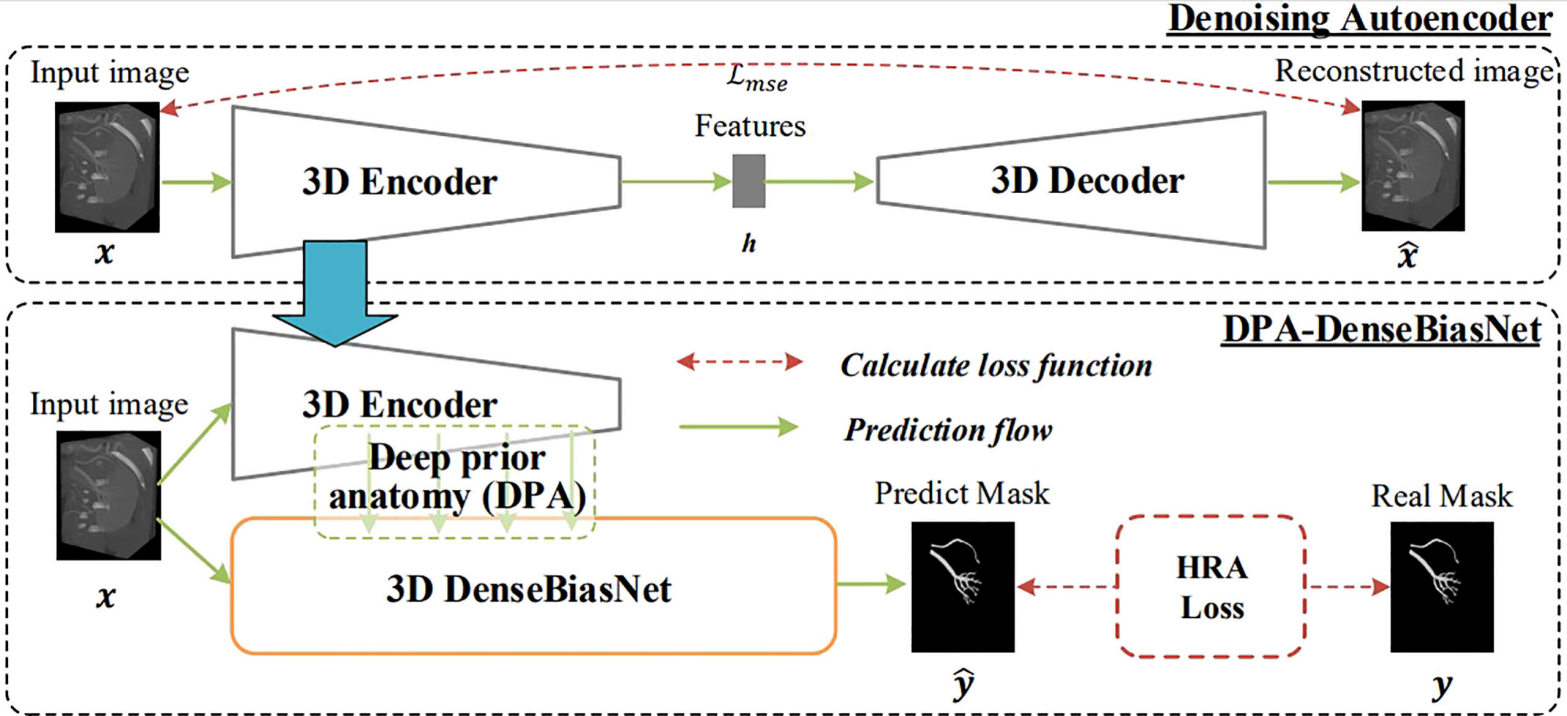
The 3D DPA-DensebiasNet framework is applied in the automatic segmentation of renal artery trees. The procedure includes two stages. Stage 1(the upper dotted box): is AE pre-training. The AE is trained by a lot of unlabeled images and DPA features are extracted. Stage 2 (the lower dotted box): extracted DPA features from AE are embedded in the DenseBiasNet system, forming the priori anatomy information, which can adapt anatomical variations. And finally, modified by HRA loss function, a precise tridimensional renal artery segmentation is achieved.

Integrating the technologies of deep-priori anatomy (DPA), dense-biased network (DenseBiasNet), and hard-region adaptation loss (HRA loss), we proposed a fine three-dimensional renal artery segmentation framework, called DPA-DenseBiasNet framework (14). The DPA-DenseBiasNet framework was based on a two-stage CNN, including (1) autoencoder (AE) network pre-training and (2) DPA features embedding and DenseBiasNet training. AE is an unsupervised neural network, which can extract anatomical features (15). In this framework, AE is applied to acquire the representation ability of anatomical features (DPA features) through a big unlabeled dataset. In stage 2, extracted DPA features from AE are embedded in the DenseBiasNet system, forming the priori anatomy information, which can adapt anatomical variations. Finally, modified by HRA loss function, a precise tridimensional renal artery segmentation is achieved (**Figure 2**). During the establishment of the DPA-DenseBiasNet framework, a total of 196 patients with 392 kidney images were recruited. Fifty-two labeled images and 236 unlabeled images were used for training, and 104 labeled images were used for testing. The DPA-DenseBiasNet was demonstrated to have high predictive accuracy in renal artery segmentation with a mean dice coefficient of 0.884.

#### Estimation of the arterial perfusion regions on the renal parenchyma

**Figure 3:**
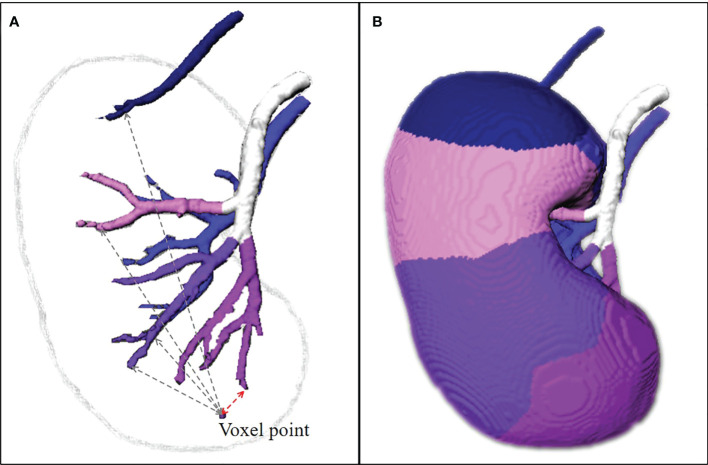
The estimation of perfusion regions. **(A)** The lobar arteries and their branches are extracted, set to the same category and marked with the same color if they branch out from the same segmental arteries. The distance transformation algorithm is used to find the closest lobar arteries or their branches for every voxel point in the renal parenchyma as its blood supply vessel, and the color of this point is marked. **(B)** All voxel points in the renal parenchyma are categorized according to their colors, and the perfusion regions of different segmental arteries are then depicted. The TKP model is finally established.

After automatic segmentation, the estimation procedure based on the two-step algorithm in C++ programming: (1) set the lobar arteries and their branches to the same category and marked with the same color if they branch out from the same segmental artery (**Figure 3A**, the arteries with the same color are the same segmental artery subtree); (2) the distance transformation algorithm is used to find the closest lobar arteries or their branches for every voxel point in the renal parenchyma as its blood supply vessel, and the color of this point is marked. All voxel points in the renal parenchyma are categorized according to their colors, and the perfusion regions of different segmental arteries are then depicted (**Figure 3B**).

By the automatic segmentation and the perfusion region estimation algorithm, the TKP model is finally established.

### Validation in animal models

#### Subjects preparation

The validation procedure in swine was approved by the Animal Use and Management Ethics Committee of Nanjing Medical University. Six domestic female pigs with 11 kidneys were recruited and the median weight was 39.5 kg (**Table 1**). All swine were intramuscularly injected with xylazine (1.5 mg/kg), atropine (0.02 mg/kg) and diazepam (10 mg) for initial anesthesia and intravenously injected with propofol (25 μg/kg/min) for maintaining anesthesia. Tracheal intubation was conducted and the right femoral artery was punctured.

**Table 1:** Patient characteristics and the TKP model information.

Variables	
Patient characteristics	
Patients, no.	131
Age, yr, mean±SD	56.3±11.4
Male, no. (%)	97 (74.0)
BMI, kg/m^2^, mean±SD	25.1±3.3
Hypertension, no. (%)	53 (40.5)
Diabetes mellitus, no. (%)	24 (18.3)
**The TKP model information**	
R.E.N.A.L score, mean±SD	6.4±1.4
**R**adius of tumor (maximal diameter), cm, mean±SD	2.5±0.8
**E**xophytic/endophytic properties, no. (%)	
≥50%	68 (51.9)
<50%	53 (40.5)
Entirely endophytic	10 (7.6)
Location relative to the polar line, no. (%)	
Entirely upper or lower polar	61 (46.6)
Lesion crosses polar line	45 (34.4)
Middle polar (>50% crosses polar line)	25 (19.1)
**N**earness to UCS/sinus, no. (%)	
≥7mm	25 (19.1)
<7mm and >4mm	64 (48.9)
≤4mm	42 (32.1)
Contact surface area (CSA), cm^2^, mean±SD	13.5±11.3
Feeding lobar artery number, no. (%)	
1	21 (16.0)
2	56 (42.7)
3	43 (32.8)
4	10 (7.7)
5	1 (0.8)
Target segmental artery number, no. (%)	
1	79 (60.3)
2	49 (37.4)
3	3 (2.3)

TKP, tridimensional kidney perfusion; BMI, body mass index; SD, standard deviation; UCS, urinary collecting system.

#### Validation procedure

**Figure 4:**
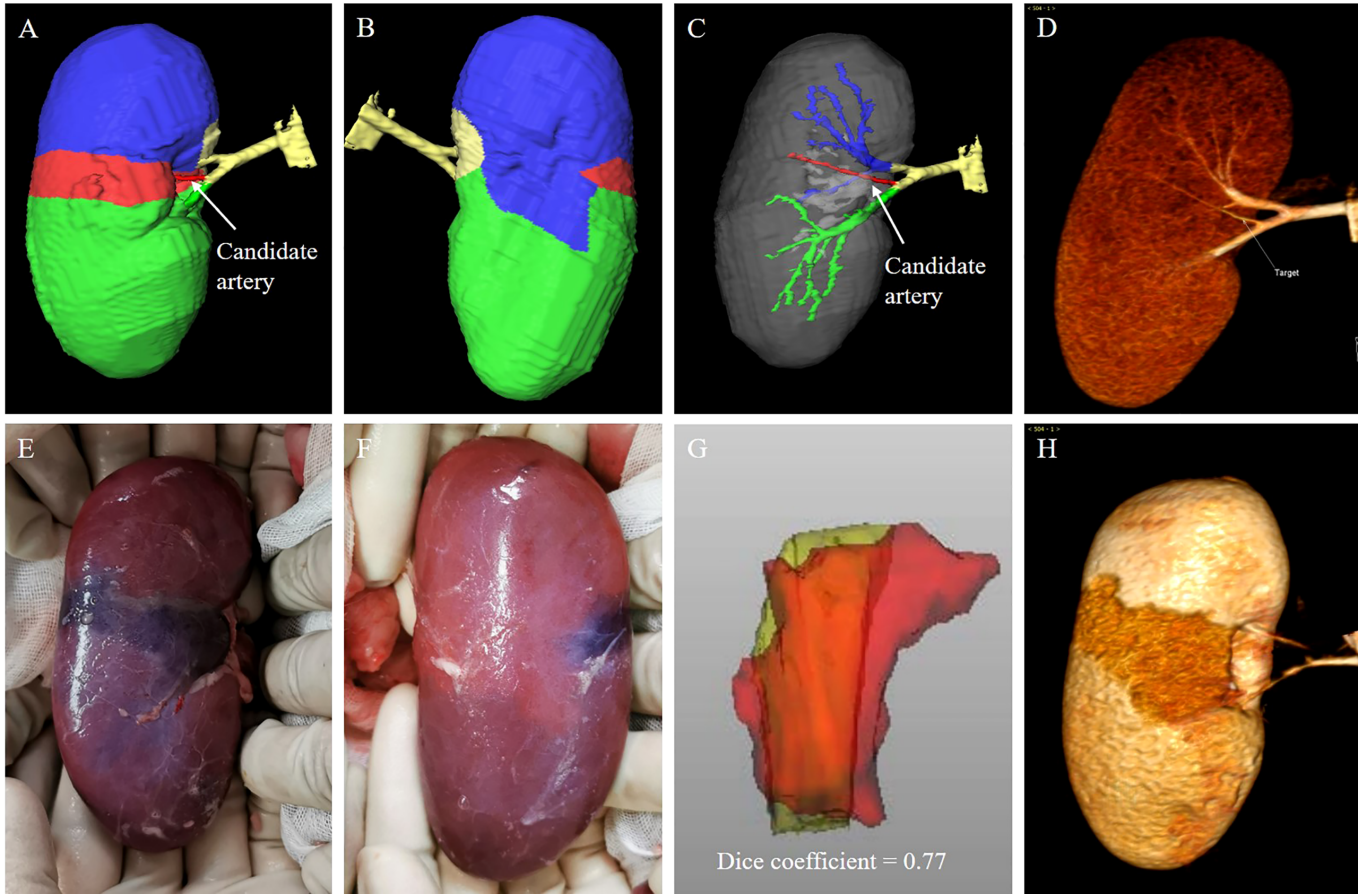
animal validation of the TKP model. **(A–D)** The TKP model of a porcine kidney is automatically established based on the first CT scan, and a candidate segmental artery is selected. **(E, F)** The ischemic line on renal parenchyma is visible and recorded after the candidate segmental artery is ligated. **(G, H)** The second CT scan is performed and the similarity between the actual ischemia region from the second CT scan and the perfusion region from the TKP model is calculated using the algorithm of dice coefficient. In this case, the dice coefficient is 0.77.

All subjects underwent contrast CT scan to establish the TKP models (**Figures 4A–D**). One candidate segmental artery in each kidney was selected and ligated with a double-strand 1/0 suture during open surgery. The ischemia region was revealed and recorded (**Figures 4E, F**). To evaluate the accuracy of the TKP model, a second contrast CT scan was performed after open surgery to present the actual ischemia region (**Figure 4H**). Using the method of dice coefficient, the actual ischemia region from the second contrast CT scan was compared with the perfusion region predicted by the TKP model (**Figure 4G**).

### Clinical application in laparoscopic PN

#### Patients preparation

Laparoscopic PN with SRAC under the TKP model’s guidance was performed in 131 patients from April 2018 to January 2020. All the recruited patients signed a written informed consent form approved by the institutional review board of Nanjing Medical University. Inclusion criteria were: 1) a single localized mass ≤4 cm (clinical T1a); 2) normal renal function (defined as creatinine clearance rate (CCR) ≥90 ml/min). All patients underwent a contrast CT scan to establish the TKP model before operation. The R.E.N.A.L scores were applied to estimate the complexity of tumors, including Radius (maximum tumor diameter), Exophytic/Endophytic, Nearness of the tumor to the collecting system, Anterior/Posterior and Location relative to the polar lines (16, 17). The contact surface area (CSA) of tumors, as another index predicting the tumor complexity (18), could be calculated by area element algorithm according to the model. Additionally, the numbers of target segmental arteries and feeding lobar arteries of tumors could be provided from the model.

#### Precise determination of the target segmental arteries

**Figure 5:**
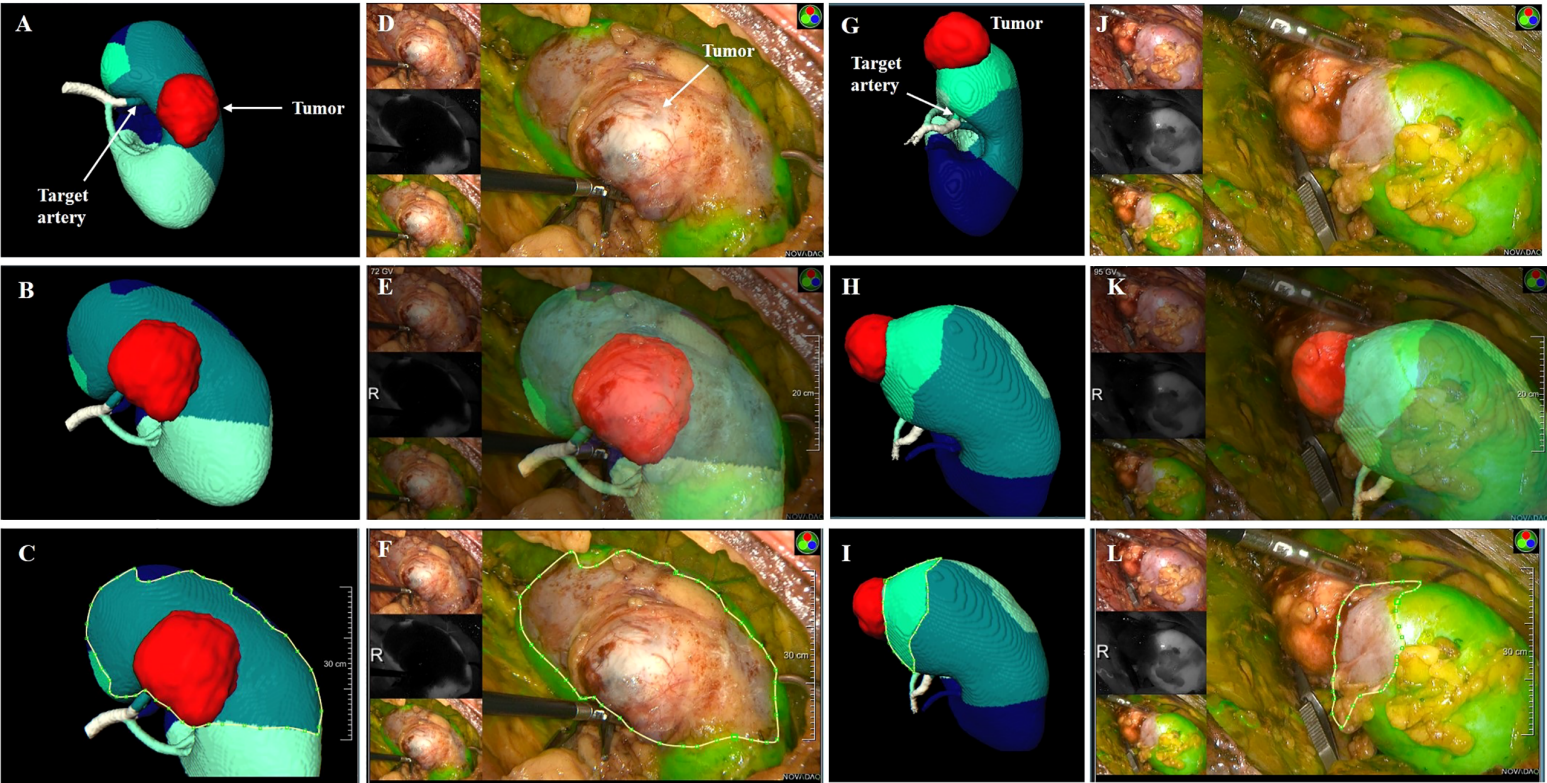
the clinical application of TKP model. **(A–F)** Case 1: a male patient with 3.8 cm tumor on the posterior part of the right kidney. The R.E.N.A.L score is 9. **(G–L)** Case 2: a female patient with 3.1 cm tumor on the upper polar of the right kidney. The R.E.N.A.L score is 5. **(A, G)** The TKP model is automatically established based on contrast CT scan and the target segmental artery is determined. **(D–F, J–L)** ICG is injected immediately after clamping, and the absence of perfusion on the renal parenchyma was confirmed with NIRF imaging. (**C** vs. **F**, **I** vs. **L**) The algorithm of dice coefficient is applied in assessing the similarity, and the dice coefficient is 0.92 and 0.81, which indicates that the ischemic region recorded in NIRF imaging is highly consistent with the perfusion region predicted in the TKP model.

In the TKP models, the tumors, segmental renal arteries and their corresponding perfusion regions were automatically presented. The target segmental arteries supplying tumors were determined by the perfusion regions wherein the renal tumors were confined (**Figures 5A, G**).

#### Surgical procedure and follow-up

All surgical procedure were conducted by the same surgeon (Pengfei Shao). During laparoscopic PN, target segmental arteries determined by TKP models were clamped by bulldog clamps. Immediately after clamping, 5 mg indocyanine green (ICG) was intravenously injected and the absence of perfusion on renal parenchyma was presented in near-infrared fluorescence (NIRF) imaging (**Figures 5D, J**). The similarity was evaluated between the absence of perfusion in NIRF imaging and the predicted perfusion region in TKP model by the method of dice coefficient. Tumor resection was conducted and the parenchymal defect was closed. Finally, the mass was retrieved to receive a pathological examination.

The follow-up period was defined as the duration from the date of operation to the date of the most recent examination. For follow-up and surveillance, abdominal plain CT scan was performed at 3 and 6 months and every 6 months. Chest CT scan and abdominal contrast CT scans were performed every 6 and 12 months, respectively.

### Statistical analysis

Categorical variables were presented as frequencies and percentages. Continuous variables were reported as mean ± SD (normal distribution) or medians and ranges (abnormal distribution). Complications were analyzed according to the Clavien–Dindo system (19). Logistic regression analysis was used to test the correlation of tumor characteristics and the number of arteries supplying the tumor. All statistical analyses were conducted using IBM SPSS v.22 (SPSS Inc., Chicago, IL, USA), and two-sided *p* < 0.05 was considered to be statistically significant. Dice coefficient was applied to evaluate the similarity of regions or volumes in two images, and high similarity was defined as dice coefficient > 0.7.

## Results

Of 11 porcine kidneys, the median number of segmental arteries was 3. After clamping, the ischemic regions were located on the upper, middle, and lower polar in three, one, and seven kidneys, respectively (**Table 1**). As shown in **Figure 4**, the boundaries of the perfusion regions in the TKP models were consistent with the ischemic lines recorded intraoperatively. It was demonstrated to have high similarity between the actual ischemia region from the post-operative CT scan and the perfusion region from the TKP model (dice coefficient = 0.82) (**Table 1**).

In clinical procedure, basic characteristics are shown in **Table 2**. There were 97 males and 34 females, aged 56.3 ± 11.4 years, with a mean body mass index of 25.1 kg/m^2^. The mean tumor size (radius of the tumor as maximal diameter) was 2.5 cm, and the R.E.N.A.L score was 6.4 ± 1.4. According to the TKP model, the CSA was 13.5 ± 11.3 cm^2^. There were 79, 49, and 3 patients with tumors supplied by one, two, and three target segmental arteries, respectively. Furthermore, subclassified by the numbers of feeding lobar arteries, there were 21, 56, 43, 10, and 1 patients with tumors supplied by one, two, three, four and five lobar arteries, respectively.

**Table 2:** Perioperative outcomes and follow-up.

Variables	
Dice coefficient (NIRF imaging vs. TKP model)	0.81 (0.72-0.94)
Operation time, min, mean±SD	100.8±11.2
Warm ischemic time, min, mean±SD	27.0±5.2
EBL, ml, median (range)	110 (40-400)
LOS after operation, days, median (range)	7 (3-17)
Post-operative complications, no. (%)	8 (6.1)
Grade 1 (hematuria not requiring intervention)	5 (3.8)
Grade 2 (hematuria requiring blood transfusion)	2 (1.5)
Grade 3a (bleeding requiring embolization intervention under local anaesthesia)	1 (0.8)
Pathology, no. (%)	
Clear cell carcinoma		112 (85.5)
Perivascular epithelioid cell tumor	7 (5.3)
Papillary renal cell carcinoma	6 (4.6)
Oxyphilic adenoma	3 (2.3)
Chromophobe renal cell carcinoma	3 (2.3)
Follow-up, mo, median (range)	21 (13-33)
Tumor recurrence and metastasis	0

NIRF, near-infrared fluorescence; TKP model, tridimensional kidney perfusion model; SD, standard deviation; EBL, estimated blood loss; LOS, length of stay.

Laparoscopic PN with precise SRAC was successfully performed under the TKP models’ guidance in all patients. The mean operation time was 100.8 min, with a mean warm ischemic time (WIT) of 27.0 min. The median estimated blood loss (EBL) was 110 ml (40 - 400 ml). There were no patients converting to main renal artery clamping, radical nephrectomy, or open surgery. No arterial bleeding or uncontrolled hemorrhage from the tumor bed occurred during tumor resection (**Table 3**). The ischemic regions recorded by ICG with NIRF imaging were highly consistent with the perfusion regions in the TKP models, with the dice coefficient of 0.81 (0.72–0.94) (**Figure 5** and **Table 3**). The median length of stay after the operation was 7 days. Postoperative complications occurred in eight (6.1%) patients, including five patients with grade 1 complication (hematuria not requiring intervention), two patients with grade 2 complication (hematuria requiring blood transfusion), and one patient with grade 3a complication (bleeding requiring embolization intervention under local anesthesia). Pathology and follow-up results were also revealed in **Table 3**. At a median follow-up of 21 months, no patient had tumor recurrence or metastasis.

**Table 3:** Validation results of TKP model in porcine models.

Variables	
Subjects/kidneys, no. Weight, kg, median (range)	6/11 39.5 (28-42)
Number of segmental arteries per kidney, median (range) Location of the perfusion regions of the candidate arteries, no. (%) Upper polar Middle polar Lower polar Dice coefficient (TKP model vs. postoperative CT scan)	3 (2-4) 3 (27.3) 1 (9.1) 7 (63.6) **0.82 (0.63-0.88)**

TKP model, tridimensional kidney perfusion model. Bold value, dice coefficient showed high similarity.

According to the number of tumor supplying arteries, subjects were divided into subgroups with one and two or more supplying segmental arteries or subgroups with 1–2 and 3–5 supplying lobar arteries. The comparisons of tumor characteristics between these subgroups are conducted in **Table 4**. Furthermore, logistic regression analysis is applied and presented in **Table 5**. On multivariate analysis, the numbers of both supplying segmental and lobar arteries strongly correlated with tumor size (OR = 5.92, *p* = 0.000 for segmental arteries and OR = 4.84, *p* = 0.002 for lobar arteries). The larger the tumor size, the more the number of supplying segmental and lobar arteries. The CSA of the tumor had correlation with the number of supplying lobar arteries (OR = 1.11, *p* = 0.014), instead of segmental arteries (*p* = 0.815). None of the correlations was found between the other tumor characteristics and the number of supplying arteries.

**Table 4:** Relationship between the tumor characteristics and the number of feeding arteries.

Variables	Target segmental artery, no.		P	Feeding lobar artery, no.		P
		1		2-3			1-2	3-5	
Patients no.	79	52		77	54	
R.E.N.A.L score	6.3 ± 1.4	6.6 ± 1.3	0.270	6.0 ± 1.3	7.0 ± 1.3	**0.000**
Radius, cm, mean ± SD	2.2 ± 0.7	3.1 ± 0.7	**0.000**	2.1 ± 0.7	3.2 ± 0.6	**0.000**
Growth pattern, no. (%)			0.212			**0.035**
Exophytic	43 (54.4)	25 (48.1)		46 (59.7)	22 (40.7)	
Mesophytic	28 (35.4)	25 (48.1)		24 (31.2)	29 (53.7)	
Endophytic	8 (10.1)	2 (3.8)		7 (9.1)	3 (5.6)	
Nearness to UCS/sinus, no. (%)			0.194			**0.001**
≥7 mm	19 (24.1)	6 (11.5)		22 (28.6)	3 (5.6)	
<7 mm and >4 mm	37 (46.8)	27 (51.9)		37 (58.1)	27 (50.0)	
≤4 mm	23 (29.1)	19 (36.5)		18 (23.4)	24 (44.4)	
Location of tumor, no. (%)			0.862			**0.019**
Entirely at the polar	38 (48.1)	23 (44.2)		42 (54.5)	19 (35.2)	
Mostly at the polar	27 (34.2)	18 (34.6)		26 (33.8)	19 (35.2)	
Mostly between polar lines	14 (17.7)	11 (21.2)		9 (11.7)	16 (29.6)	
CSA, cm^2^, mean ± SD	9.2 ± 8.2	20.0 ± 12.4	**0.000**	7.6 ± 5.8	22.0 ± 12.0	**0.000**

SD, standard deviation; UCS, urinary collecting system; CSA, contact surface area. Bold value, p value < 0.05.

**Table 5:** Logistic regression analysis on the numbers of target segmental arteries and feeding lobar arteries.

Variables	Univariate analysis			Multivariate analysis		
	OR	95% CI	P	OR	95% CI	P
**TSA no. (2-3 vs 1)**						
Radius	6.43	3.32-12.43	**0.000**	5.92	2.29-15.30	**0.000**
CSA	1.10	1.06-1.15	**0.000**	1.01	0.95-1.07	0.815
**FLA no. (3-5 vs 1-2)**						
Radius	11.88	5.26-26.85	**0.000**	4.84	1.76-13.29	**0.002**
Growth pattern			**0.037**			0.326
Exophytic	ref					
Mesophytic	2.53	1.20-5.31	**0.014**			
Endophytic	0.90	0.21-3.80	0.882			
Nearness to UCS/sinus			**0.004**			0.784
≥7 mm	ref					
<7 mm and >4 mm	5.35	1.45-19.72	**0.012**			
≤4 mm	9.78	2.53-37.80	**0.001**			
Location of tumor			**0.023**			0.318
Entirely at the polar	Ref					
Mostly at the polar	1.62	0.72-3.60	0.241			
Mostly between polar lines	3.93	1.48-10.47	**0.006**			
R.E.N.A.L	1.76	1.31-2.36	**0.000**			0.495
CSA	1.20	1.12-1.29	**0.000**	1.11	1.02-1.20	**0.014**

TSA, target segmental artery; FLA, feeding lobar artery; CSA, contact surface area; UCS, urinary collecting system; OR, odd ratio; CI, confidence interval. Bold value, p value < 0.05.

## Discussion

Traditionally, DSCT angiography was utilized to reveal the three-dimensional hilar vasculature during SRAC procedure (5). In DSCT angiography, the target arteries were determined manually, leading to the underestimation of artery branches feeding both tumor and the surrounding normal tissue (7). So measurement bias, insufficient clamping, arterial bleeding or even converting to the main artery clamping during resection might occur. For a more efficient and precise SRAC technique, the TKP model was established using a homemade CNN technology, becoming an automatic tool in the surgical strategy-making of the SRAC during nephron-sparing surgery (14). ICG with NIRF imaging, as an empirical technique of fluorescence guidance (20), was introduced in this study to delineate the real ischemic area after clamping. Our results confirmed that the perfusion regions predicted in the TKP model were highly consistent with the real ischemic area in NIRF imaging during operation. In this study, under the guidance of the TKP models, all surgical procedures were performed successfully, and there was no occurrence of uncontrolled bleeding during tumor resection.

Recently, by constructing various models, researchers have been exploring the navigation technique in PN with SRAC. Ukimura et al. established a 3D model that could present opaque tumors and renal arterial trees by making the renal parenchyma semitransparent (21). By manually segmenting kidney shape, vasculature, collecting system, and tumor, Porpiglia proposed a hyperaccuracy 3D model (22), which used an augmented reality (AR) technique to guide surgeons during operation (23). Additionally, the goal of purely automatic segmentation of different organs and even renal artery trees using CNNs was achieved (24–26). We had developed a series of novel CNNs, including 3D_FCN_PPM and DPA-DenseBiasNet, providing a precise segmentation of kidney, tumors, renal arteries and their branches (distal to interlobar arteries) (13, 14). These homemade CNNs, along with the distance transformation algorithm, made the establishment of the TKP model fully automated. And the automatic procedure could reduce the manual workload of delineation in the radiological process and significantly improve the efficiency of the preoperative plan of laparoscopic PN with a precise SRAC technique.

Using CNN techniques, automatic segmentation could be accurate to distal interlobar arteries, facilitating a more precise estimation of the arterial perfusion regions. In the future, in combination with the AR technique, it is expected that the TKP model could be implanted into the surgery console and become visual and synchronous. It is beneficial for improving the accuracy and efficacy of SRAC technique and tumor resection.

Leslie and his colleagues presented the CSA as a novel parameter to predict the complexity of renal tumors (18). In our study, tumor size and CSA strongly correlated with the number of feeding arteries. Larger CSA was accompanied by more feeding lobar arteries, instead of the target segmental arteries. In the future, the number of lobar arteries feeding the tumor is expected to predict renal tumor complexity and become an indicator in the scoring system to evaluate the difficulty of PN.

This study is not devoid of limitations. Firstly, we still lack a multi-center research. Secondly, we lack a randomized controlled study to compare the TKP model and the traditional DSCT angiography model since the former is a newly developed technology.

Notwithstanding these limitations, the TKP model was more than satisfactory because of the hyperaccuracy verified during operation. It is expected to become a comprehensive tool with multiple functions, such as preoperative assessment of tumor complexity, automatic planning of surgical strategy and real-time navigation of selective clamping and tumor resection.

## Conclusions

Using the CNN technique, the TKP model is developed to automatically present the renal artery trees and precisely delineate the perfusion regions of different segmental arteries. The guidance of the TKP model is feasible and effective in nephron-sparing surgery.

## Data availability statement

The raw data supporting the conclusions of this article will be made available by the authors, without undue reservation.

## Ethics statement

The studies involving human participants were reviewed and approved by the institutional review board of Nanjing Medical University. The patients/participants provided their written informed consent to participate in this study.The animal study was reviewed and approved by the Animal Use and Management Ethics Committee of Nanjing Medical University. Written informed consent was obtained from the individual(s) for the publication of any potentially identifiable images or data included in this article.

## Author contributions

SZ, GY, PS and ZW contributed to the study conception and design. The acquisition of data was performed by SZ, XZ, YH and YX. Data analysis and interpretation: GY, XZ, YH, YX. The first draft of the manuscript was written by SZ, JQ, YH and PS, and critical revision of the manuscript was performed by SZ, JL, PL and PS. Supervision of whole procedure was performed by JL, PS and ZW. All authors read and approved the final manuscript.

## Conflict of interest

The authors declare that the research was conducted in the absence of any commercial or financial relationships that could be construed as a potential conflict of interest.

## Publisher’s note

All claims expressed in this article are solely those of the authors and do not necessarily represent those of their affiliated organizations, or those of the publisher, the editors and the reviewers. Any product that may be evaluated in this article, or claim that may be made by its manufacturer, is not guaranteed or endorsed by the publisher.
